# Characteristics of Home-Visit Nursing Agencies That Closed after the 2012 Fee Revision for Home-Visit Nursing Services: A Nationwide Panel Data Analysis in Japan

**DOI:** 10.3390/ijerph18189820

**Published:** 2021-09-17

**Authors:** Masayo Kashiwagi, Noriko Morioka

**Affiliations:** Graduate School of Health Care Sciences, Tokyo Medical and Dental University, Tokyo 113-8519, Japan; morioka.gh@tmd.ac.jp

**Keywords:** agency closure, home care agencies, home-visit nursing agencies, home health nursing, home-visit nursing, long-term care, long-term care insurance system, market competition, panel data

## Abstract

Despite the 2012 fee revision raising fees for home-visit nursing services to increase their supply in Japan, 300 to 500 home-visit nursing agencies (VNA) are still being closed annually. This study aims to identify the regional and organizational characteristics of the VNAs that closed after the 2012 fee revision. A longitudinal observational study was conducted using nationwide panel data of VNAs from 2014 to 2017 (N = 6496). Multiple logistic regression models stratified by years of operation were used for the analysis. We identified 821 closed agencies (12.6%). In this study, many important factors related to VNA closures were found. In the less than three years group, there were regional factors (lower aging rate and larger number of clinics) and an organizational factor (higher proportion of users under 40 years of age). In the 3–14 years group, there was a regional factor (larger number of clinics) and organizational factors (smaller number of FTE nurses, smaller number of users per FTE nurse, and smaller number of medical care types that can be provided). In the over 15 years group, there was an organizational factor (smaller number of FTE nurses). The findings provide valuable insights for policymakers in avoiding VNA closures.

## 1. Introduction

Aging populations and increasing demand for long-term care are globally recognized as important policy issues [1]. Japan has the world’s highest proportion of individuals aged 65 years or older. By 2019, 28.4% of Japan’s population had already reached 65 years of age [2]. The domestic demand for medical and nursing care is expected to increase in 2025. By then, all of Japan’s baby boomers will have become 75 years or older. Therefore, enhancing professional nursing care services that support older adults’ in-home care will become essential.

The Japanese government introduced a public long-term care insurance system (LTCI) in 2000. With this, Japan shifted from the traditional family care to social care for the frail elderly. The LTCI operates as a social insurance system. Everyone aged 40 years and older pays premiums. Moreover, all people aged 65 years and older (and aged 40–64 years, if the need arises from an aging-related disease) is eligible for benefits. Many services are covered. There are home services such as home-visit care by care workers. They visit their clients’ homes to aid them with bathing, ablutions, and household chores, among others. Likewise, there are daycare services such as physical rehabilitation, meals, and bathing assistance at a facility. Finally, there are also home-visit nursing services being provided [3]. Under the LTCI, the users are theoretically free to choose their service mix and providers. However, the care managers are actively involved in the care plans and service arrangements [3] The local government licenses and supervises the service providers. Moreover, the national government sets the fees for each service and revises them every 3 years.

The most radical reform related to the LTCI allowed for-profit organizations to enter the in-home care service market. This was undertaken to increase the supply of services [4]. The reform has delivered some positive results. By 2010, a decade after the LTCI was introduced, the number of home-visit care agencies was approximately 3.6 times higher. Particularly, care workers visited their clients’ homes more to aid them with bathing, ablutions, and household chores, among others. The number of the daycare service agencies that provide services, such as physical rehabilitation, meals, and bathing assistance at a facility, has also increased by approximately three times more than the number before the LTCI [5].

Meanwhile, the home-visit nursing agencies (VNA) market has not progressed. The Japanese government has responded to home healthcare services’ future growing demand by promoting home-visit nursing services as major service. Since the LTCI began, there have been many positive revisions focused on securing a sufficient number of VNAs. This includes introducing service provision systems that can add fees. However, the number of VNAs has remained at about 5000 after a decade with the LTCI [5]. According to recent studies, the annual VNA profit rate was 4.2% in 2020 [6]. Additionally, most VNAs have few staff. In 2011, the average numbers of nursing staff and other staff such as rehabilitation professionals per agency were 4.2 and 1.7, respectively [5]. A prior study reported that approximately 20% of the VNAs were unprofitable, particularly the small-scale ones [7]. Japan’s situation may have resulted in the stagnation of the number of VNAs.

The Japanese government raised the fee-for-service for home-visit nursing in 2012 to improve this situation. This was a simultaneous revision with medical insurance. The service fee rise contributed to approximately 1000 to 1300 of the VNAs that have entered the market annually since 2014 [5]. Conversely, 300 to 500 equivalents of around 10% of all the agencies have closed annually. The National Association for Visiting Nurse Service reported 303 VNA closures nationwide in 2014, 425 in 2015, 462 in 2016, 482 in 2017, and 534 in 2018. This number has continued to grow since then [8]. In particular, the increase in the number of VNAs has slowed down in recent years. The total number of VNAs in Japan has only slightly increased by approximately 1.5 times (from 6998 to 10,884) from 2014 to 2018 [5]. The 2012 revision somehow caused market competition among the VNAs. This may have resulted in user competition among the increased number of agencies. Consequently, some agencies that were unable to attract users may have been closed. It is possible that a continued increase in VNA closures may lead to an inability to meet the future demands for home-visit nursing care. Identifying the characteristics of the VNAs that closed after the 2012 revision has not been undertaken in Japan, even though it is necessary to improve the situation.

There have been a few reports on the factors related to VNA closure. A study reported that proprietary, freestanding organizations and those having a smaller total number of staff are related to home health agency closures [9]. Moreover, another study reported that smaller, newer, freestanding, and operating organizations with more visit-intensive practice styles in the market environment are related to home health agency closures [10]. Although the results showing the relation of smaller organizations with agency closure were almost concordant, the VNA scale in the United State is larger than that in Japan. Moreover, the public insurances make the home-visit nursing services provision unique in Japan. Specifically, service use is generally arranged by a care manager [11,12,13,14]. Moreover, written directions issued by a physician is required to start the home-visit nursing services [15]. These factors need to be controlled to identify the factors related to VNA closures. Concurrently, some studies have examined factors related to nursing facility closures, such as nursing homes or care homes. Studies identifying factors related to the nursing home closures found that the following facilities indicated a significantly higher likelihood of closure: facilities located in markets with high levels of competition [16,17,18], those which are hospital-based [19], those with chain members [16,17], those which reduced the number of beds [19], those that were newer [17], and those with losses of 5% or more [19]. These prior studies suggest that both organizational factors (e.g., agency size, year of operation, and operating organizations) and regional factors (e.g., competition) may influence VNA closures.

The present study seeks to identify the characteristics of the VNAs that closed after the 2012 fee revision. Using a nationwide panel data analysis in Japan, we first identified the agencies operating in fiscal year 2014 that subsequently closed in the next three years (fiscal years 2015–2017). Second, we clarified the regional and organizational factors associated with VNA closures by year of operation using multiple logistic regression analysis.

## 2. Materials and Methods

### 2.1. Design and Data Collection

This study employed a longitudinal observational study design. We constructed a panel of nationwide administrative data for VNAs in Japan covering the years 2014, 2015, 2016, and 2017. We used 2014 as a baseline year because VNAs have been entered and closed rapidly since 2014. The follow-up period was until 2017 to conform the closed agencies.

We collected data from the “Information Publication System for Long-term Care insurance services database (LTC-DB)” by the Ministry of Health, Labor, and Welfare [20]. The LTC-DB started in April 2006 under the LTCI. It is an online mechanism by which prefectures published information on home care service agencies including VNAs, and long-term care facilities throughout Japan. It sought to help those attempting to access LTCI services choose an agency [20]. 

**Figure 1:**
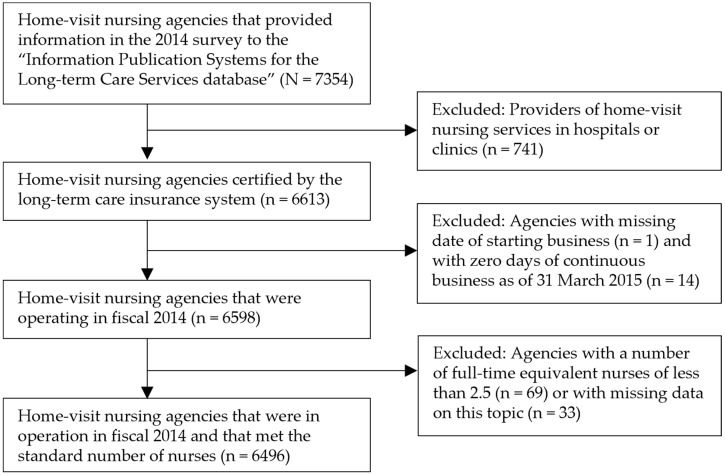
Flow chart of the process of selecting home-visit nursing agencies.

The data included information on the opening date, location, agency ownership, service content, number of users, and number of staff. We collected published information on the VNAs in Japan from 2014 to 2017 in this study. Particularly, we created a panel database (N = 7354) combining VNA information for each year according to their business operator ID. The following were excluded from the database: hospitals or clinics providing home-visit nursing services; with missing information on the date they started their business; agencies that were registered but have failed to commence their operations as of March 31, 2015; agencies with less than 2.5 full time equivalent (FTE) nurses, and agencies with missing figures for FTE nurses. Finally, 6496 agencies were identified (Figure 1). 

According to an annual nationwide government survey on nursing care insurance providers, the number of VNAs was 7903 in 2014 [5]. Furthermore, the coverage rate of the agencies analyzed in the current research was 82.2%.

This study exclusively used published data only. As such, no personal data have been included.

### 2.2. Measurements

#### 2.2.1. Dependent Variables

The dependent variable for this analysis was the binary variable of “closed” or “not” of VNAs from 2015 to 2017. “Closed” is defined as the closing of a VNA and the discontinuance of the provision of home-visit nursing care. 

**Figure 2:**
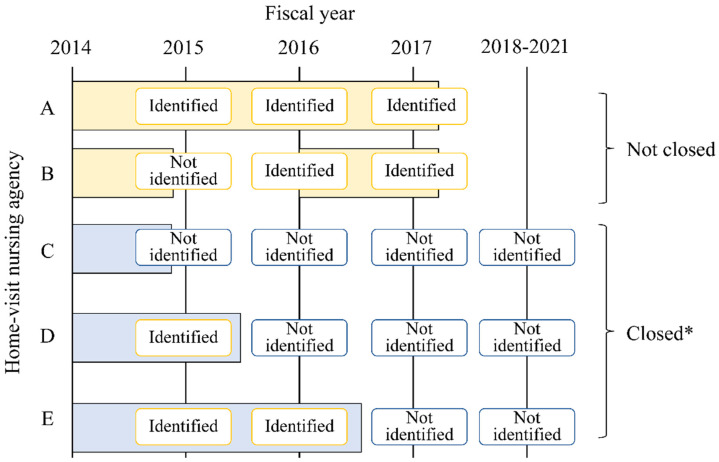
Identifying closed home-visit nursing agencies. * We confirmed whether their information appeared in more recently published datasets using information from the LTC-DB published online in 2018, 2019, and 2020.

The local government has information on the closed VNAs. However, the list of closed VNAs has not been made public. Thus, in this study of the VNAs included in the 2014 publication of information through the LTC-DB, those that did not have the corresponding information between 2015 and 2017 were designated as “closed”. However, there was the possibility that some agencies considered as “closed” may not have registered their agency’s information for the years included in this investigation. Accordingly, we confirmed whether their information appeared in more recently published datasets using the information from the LTC-DB published online in 2018, 2019, and 2020 [20] (see Figure 2 below).

#### 2.2.2. Independent Variables

The independent variables were potentially related factors from prior studies [7,9,10,11,12,13,14,15,16,17,18,19]. They were classified into “regional factors”, based on where the VNA is located and “organizational factors”.

##### A. Regional Factors

Regional factors included the municipality, where the VNA has a “percentage of older adults 65 years or over”, and has an “inhabitable area (square km)” in the municipality where the VNA is located. In addition, we used the “number of VNAs per 100,000 population” and the “number of clinics per 100,000 population” that were an index of the market competition in the municipality, where the VNA is located. These variables were extracted from a statistical database published by the government [21].

##### B. Organizational Factors

Organizational factors included the following: “years of operation as of 31 March 2015”, calculated from the opening date, “for-profit organization” (yes or no), “medical organization” (yes or no), “attached to a hospital” (yes or no), “provision of care management services under the LTCI within the same organization” (yes or no), “number of medical institutions issuing home-visit nursing orders”, “number of FTE nurses”, “percentage of nurses with less than three years of experience in the VNA”, “percentage of full-time nurses”, “number of users per FTE nurses”, “change in the number of nurses in 2013” (decrease, no change, increase), and “percentage of rehabilitation professionals among home-visit staff”. In addition, the service provision systems that can add fees to the LTCI were also designated as independent variables. These include: “provision system of emergency home-visits” (yes or no), “provision system of home-based palliative care” (yes or no), “provision system of specialized medical care 1” (yes or no; i.e., special medical management, such as tracheal cannula, malignant tumors, tracheotomy, or indwelling catheters), “provision system of specialized medical care 2” (yes or no; i.e., undergoing abdominal dialysis, hemodialysis, oxygen therapy, central venous nutrition, etc., at home), “enhanced service delivery system” (yes or no; e.g., employing a certain amount of staff with a certain level of experience), “cooperative discharge guidance with hospitals” (yes or no; i.e., where hospitals and VNAs collaborate to provide discharge guidance before patients are discharged), and “number of types of medical care that can be provided”. Moreover, “percentage of users under 40 years”, who are not covered by the LTCI and the “percentage of users with LTCI-certified care need level of four or higher” (ranging from levels one to five, with care need levels of four and five referring to bedridden care receivers) were also used as independent variables. These variables were collected from the LTC-DB [20].

### 2.3. Statistical Analysis

First, the data were examined using descriptive statistics. Thereafter, agencies were stratified into the three categories according to years of operation (<three years, three-14 years, ≥15 years) as of 31 March 2015 to identify the characteristics of the closed agencies. Less than three years of operation means that the agencies were opened after 2012. Moreover, less than 15 years of operation means that the agencies were opened after the LTCI’s introduction. Concurrently, more than 15 years meant that the agencies were opened before the LTCI’s introduction. The bivariate analyses using chi-squared tests or the Wilcoxon rank sum test were then conducted to determine the association among the independent variables. These were the regional and organizational factors and dependent variable, and the VNA being “closed” or “not closed”. Finally, multivariable logistic regression analyses were performed by year of operation using the binary variables of “closed” or “not” as dependent variables. All independent variables were applied using the forced-entry method after confirming the absence of multicollinearity. The relationship’s strengths were explained using the adjusted odds ratios (AOR) and 95% confidence intervals (95% CIs). The Hosmer–Lemeshow test was used to test the model’s goodness-of-fit. An alpha level of 0.05 was employed for all statistical tests. We used Statistical Analysis System version 9.4, for Windows (SAS Japan Inc., Tokyo, Japan) for the analysis.

## 3. Results

### 3.1. Basic Characteristics of the Study’s Population and Closed Agencies

Table 1 shows the study sample’s basic characteristics. Of the 6496 agencies operating in 2014, 821 were closed from 2015 to 2017. It was found that 54.7% of the closed VNAs were for-profit organizations. Their median years of operation (as of 31 March 2015) was four years. In contrast, 57.1% of the closed VNAs provided care management services within the same organization. They had a median of 3.2 FTE nurses. The median number of users per FTE nurses was 8.0. They had no enhanced service delivery.

Table 2 shows the closure rate and number of VNAs by their year of operation. The closure rate by years of operation was 23.1% (n = 342) in agencies operating for less than three years, 12.3% (n = 307) in agencies operating for 3–14 years, and 6.8% (n = 172) in agencies operating for over 15 years. Regarding the agency’s ownership, 54.7% of the closed agencies were for-profit organizations (n = 449).

### 3.2. Factors Related to Home-Visit Nursing Agency Closure by Years of Operation

The results of the multivariable logistic regression analysis are shown in Table 3 (the results of the bivariate analysis are shown in the Appendix A). AOR and 95% confidence intervals (95% CIs) for the regression model are also presented. The following factors were related to agency closures: 

In the group of agencies operating for less than three years, the regional factors significantly associated with VNA closures were: a lower percentage of older adults aged 65 years or older (AOR: 0.963, 95%CI: 0.935–0.991), a smaller number of VNAs per 100,000 population (AOR: 0.991, 95%CI: 0.978–0.999), and a larger number of clinics per 100,000 population (AOR: 1.004, 95%CI: 1.001–1.007). The organizational factor significantly associated with VNA closures was: a higher percentage of users under 40 years of age (AOR: 1.019, 95%CI: 1.005–1.034).

In the group of agencies operating for 3–14 years, the regional factors significantly associated with VNA closures were: a smaller number of VNAs per 100,000 population (AOR: 0.997, 95%CI: 0.991–1.000) and a larger number of clinics per 100,000 population (AOR: 1.003, 95%CI: 1.001–1.006). The organizational factors significantly associated with VNA closures were: a smaller number of FTE nurses (AOR: 0.897, 95%CI: 0.827–0.966), a smaller number of users per FTE nurse (AOR: 0.952, 95%CI: 0.927–0.976), and a smaller number of types of medical care that can be provided (AOR: 0.940, 95%CI: 0.894–0.988).

In the agencies operating for over 15 years, the organizational factors significantly associated with VNA closures were: a smaller number of FTE nurses (AOR: 0.848, 95%CI: 0.755–0.942) and having a provision system for emergency home-visits (AOR: 1.869, 95%CI: 1.023–3.571).

## 4. Discussion

This study presents the characteristics of closed agencies. Moreover, it shows that the closure rate of newly opened groups, after the 2012 revision, was high. Our results suggest that the 2012 fee revision may have influenced VNA closures. The drastic VNA market exiting after the fee revision is consistent with the prior study reporting the changing payment system’s impact (the Interim Payment System and Prospective Payment System) [9,10]. It is important to state that the factors related to agency closure varied across the three groups of the agencies’ years of operation (newly opened group, opened group after the LTCI’s introduction, and opened before the LTCI’s introduction). 

In the newly opened group, our results showed that almost a quarter of the agencies closed over three years, despite the increase in the fee with the 2012 revision. This occurred after less than three years of being in the operation group. It is important to note that the VNA located in a small number of VNAs per population and a large number of clinics per population were associated with the closure. Prior studies showed that facilities located in markets with high levels of competition are more likely to close [16,17,18]. However, the competitor was unclear. In Japan, home-visit nursing services are provided by VNAs, hospitals, and clinics. We expected that competition has occurred among the VNAs after the 2012 revision. Contrary to this expectation, competition has occurred between VNAs and clinics. To the best of our knowledge, this is the first study demonstrating such a relationship between VNAs and clinics. On the other hand, home-visit nursing services from hospitals and clinics have decreased in recent years [5]. Several studies in the United States have shown a decrease in the likelihood of receiving home care. Similarly, they also showed a decrease in the number of visits patients receive after the Balanced Budget Act [22,23]. Another study also reported on the competition market, showing that the number of visits had dropped significantly [24]. There is concern regarding the decrease in the supply of home-visit nursing services in municipalities with large numbers of VNA closures. Therefore, further studies on VNA closures’ impact on service supply are needed. Additionally, our results showed that the organizational factor “a high percentage of users under 40 years of age” was associated with the closure. More than half of the home-visit nursing users are older adults aged 65 years or above [25]. These results suggest that market research was insufficient upon entering the market. Our findings imply that in establishing a VNA, it is important to enter the market in an area with a small number of VNAs and to identify and analyze the market’s needs and its size. Market research should include the number and percentage of older adults over 65 years of age in the area. They must likewise touch upon whether the VNA can ensure a sustainable number of home-visit nursing service users.

In the group that opened after the LTCI’s introduction (3–14 years of operation group), agency closure was associated with a regional factor (a large number of clinics per population) and organizational factors (a small number of FTE nurses, a small number of users per FTE nurse, and a small number medical care types that can be provided). Similar to the previous group, the results suggest that even if a VNA is established in a region with a large number of clinics, it may be difficult to attract new users in the region. Additionally, organizational factors (e.g., a small number of FTE nurses, a small number of users per FTE nurse, and small number of types of medical care that can be provided) were also associated with closure. Our study’s results suggest that closed agencies were a hindrance to continuously ensuring new users. A prior study has shown that the number of nursing staff and patients per nursing staff was associated with profitability [7]. This means that the predictors of agencies’ unprofitability are related to higher closure rates. According to national statistics, the labor cost ratio is around 80% per agency [6]. In other words, the VNA industry is labor-intensive. This study’s results suggest that it is necessary to increase the size, the number of users per nurse, and the provision of various medical care to ensure continuity of business operations. Therefore, it may be necessary not only to revise the service fee, but also support the system to continue operations at the municipality level to increase the number of VNAs. These include expert management consulting for VNAs with a closure risk, and training on the provision of medical care in home, among other services.

Finally, it should be noted that in the group that was opened before the LTCI’s introduction (over 15 years of operation group), we identified that only organizational factors (e.g., having a provision system of emergency home-visit nursing care and a small number of FTE nurses) were associated with closure. This may be due to the difficulty in securing nurses as the number of VNAs have increased rapidly after the 2012 fee revision. Moreover, 20 years have passed since the LTCI’s introduction, and the number of older nurses is increasing. These findings also suggest that the aging nursing-workforce that supported many agencies even before the LTCI’s implementation may have affected the VNA closures. The agencies providing emergency home-visit nursing and those having a few FTE nurses will have difficulty in continuing to operate unless they can secure younger nurses. The Japanese Nursing Association, Japan Visiting Nursing Foundation, and the National Association for Visiting Nurse Service have established nursing education programs allowing new graduates to become visiting nurses. Moreover, these programs increase the number of new graduate nurses working at VNAs [26]. However, a very small percentage of VNAs hire new graduates. Therefore, for VNAs that have been operating before the LTCI’s introduction, the manner and procedure by which VNAs resolve the aging nursing workforce issue is crucial when endeavoring to support the LTC demand in Japan. Similarly, how they secure younger staff to take on home-visit nursing responsibilities is likewise an important issue that must be addressed.

This study has some limitations. First, it is possible that a VNA that has moved its location was classified as “closed”. When an agency moves to another municipality, a notification of the operation’s cessation is submitted to the local government prior to the relocation. After moving, a new application to open an agency is made with the local government and a new business ID is registered. Therefore, identifying the location where the VNA has moved to is difficult when using only the database in the present study. The second limitation is that, although the nationwide coverage of the agencies listed in the 2014 “Information Publication Systems for the long-term Care Services” was high at 82.2%, the coverage of the Yamanashi Prefecture (4.0%), Ibaragi Prefecture (20.8%), Chiba Prefecture (34.7%), and Kyoto Prefecture (42.4%) was below 50% in each region. The area definition of the regions where the VNAs are located were based on city-level data. This is likely to have a minimal effect on the results. Thus, caution should be exercised when applying the results to these prefectures. The third limitation is that this study used a retrospective observational study design. Thus, the real reason for each VNA’s closure was not available. Further studies, specifically interview studies, are needed to clarify the real reason for VNA closures.

Despite these limitations, this is the first study to identify the number and rate of VNA closures from 2015 to 2017 after the 2012 service fee revision. It is also the first study to show closure-related factors by the number of years of operation. From our results, it can be assumed that the 2012 service fee revision did not contribute much to the increase in the number of VNAs. In addition, our results suggest the need for policymaking that focuses on the balance between the supply and demand for home-visit nursing services in each municipality. It likewise stresses the importance of securing nurses rather than the number of VNAs.

## 5. Conclusions

The results showed that the VNA closure rate was the highest among agencies operating for less than three years. The agency closure-related factors varied across the agencies’ years of operation. The closure characteristics of agencies that operated for less than three years were as follows: a low percentage of older adults over 65 years, a small number of VNA per 100,000 population, a large number of clinics per 100,000 population, and a high proportion of users under 40 years of age. The reduction in the overall number of VNAs, despite the increase in the fee with the 2012 revision, was mainly explained by the fact that newly opened agencies entering the market in response to the rising fees were quickly heading toward closure. This was due to both insufficient market research upon entering and market competition with the clinics providing home-visit nursing services. The findings showed that the characteristics associated with the closure of agencies that had been operating between 3 and 14 years were a large number of clinics per 100,000 population, a small number of FTE nurses, a small number of users per FTE nurse, and a small number of types of medical care that can be provided. For the group of agencies that had been operating for over 15 years (i.e., those that were established prior to LTCI’s introduction), the agency closures’ characteristics were having a provision system of emergency home-visit nursing and a small number of FTE nurses. 

These findings suggest that the government should have considered not only promoting new VNA’s entry by raising the service fee, but also showing the necessary amount of home-visit nursing services by VNAs based on the demand for home-visit nursing care services in the municipality level. This research needs undertaken in response to meeting the near-future demand increases for home-visit nursing. Furthermore, it is necessary to make policies avoiding the closure of VNAs, such as policies on conducting marketing surveys and securing nurses.

## Figures and Tables

**Table 1:** The basic characteristics of the study population, total, and closed home-visit nursing agencies.

	Total		Closed	
	(n = 6496)		(n = 821)	
	Median ± 25–75 percentile or number of subjects (%)			
A. Regional factor				
Percentage of older adults 65 years old or over	26.7	23.5–31.2	26.0	23.0–30.3
Inhabitable area (square km)	2229.7	823.9–7417.9	3031.3	930.3–8329.9
Number of VNAs per 100,000 population	7.0	5.2–9.6	7.0	5.3–10.1
Number of clinics per 100,000 population	77.8	64.9–93.5	79.3	65.9–94.9
B. Organizational factor				
For-profit organization				
Yes	2594	(39.9)	449	(54.7)
No	3901	(60.1)	372	(45.3)
Medical organization				
Yes	2080	(30.2)	203	(24.7)
No	4415	(68.0)	618	(75.3)
Years of operation as of March 31, 2015	10.3	3.2–15.9	4.0	1.0–12.7
Provision of care management services				
Yes	4570	(29.6)	469	(57.1)
No	1926	(70.4)	352	(42.9)
Number of medical institutions issuing home-visit nursing orders	19	9–32	11	3–23
Number of FTE nurses	4.0	3.0–5.6	3.2	2.7–4.5
Percentage of nurses with less than three years of experience in the VNAs	33.3	12.5–62.5	40.0	0.0–85.7
Percentage of full-time nurses	66.7	42.9–85.7	66.7	42.9–100.0
Number of users per FTE nurses	11.8	7.7–16.5	8.0	3.4–14.0
Change in the number of nurses in 2013				
Decrease	924	(14.2)	86	(10.5)
No change	3680	(56.7)	522	(63.6)
Increase	1892	(29.1)	213	(25.9)
Percentage of rehabilitation professionals among home-visit staff	1.3	0.0–30.0	0.0	0.20.0
Provision system of services				
Emergency home-visits				
Yes	5482	(84.4)	222	(27.0)
No	1014	(15.6)	599	(73.0)
Home-based palliative care				
Yes	5252	(80.8)	535	(65.2)
No	1244	(19.2)	286	(34.8)
Specialized medical care 1				
Yes	5638	(86.8)	598	(72.8)
No	858	(13.2)	223	(27.1)
Specialized medical care 2				
Yes	5599	(86.2)	583	(71.0)
No	897	(13.8)	238	(29.0)
Enhanced service delivery				
Yes	3172	(48.8)	228	(27.8)
No	3324	(51.2)	593	(72.2)
Cooperative discharge guidance with hospitals				
Yes	4839	(74.5)	486	(59.2)
No	1657	(25.5)	335	(40.8)
Number of types of medical care that can be provided	12	10–13	11	6–13
Percentage of users under the age of 40 years	1.1	0.0–4.5	0.0	0.0–4.2
Percentage of users with care need level of four or higher	34.2	25.0–45.5	33.3	22.6–48.6

**Table 2:** Number of closures and the closure rate of home-visit nursing agencies by years of operation.

Years of Operation	Total (n = 6496)	Closed (n = 821)		Not Closed (n = 5673)	
		n	%	n	%
<3 years	1479	342	23.1	1137	76.9
3–14 years	2490	307	12.3	2183	87.7
≥15 years	2527	172	6.8	2355	93.2

**Table 3:** Multivariable logistic regression analysis on the factors related to the closure of home-visit nursing agencies by years of operation.

Variables	<3 Years of Operation (n = 1195)			3–14 Years of Operation (n = 2356)			≥15 Years of Operation (n = 2450)		
	AOR	(95%CI)	*p*	AOR	(95%CI)	*p*	AOR	(95%CI)	*p*
A. Regional factor									
Percentage of older adults 65 years old or over	0.963	(0.935–0.991)	0.010	0.999	(0.976–1.023)	0.957	1.006	(0.978–1.034)	0.672
Inhabitable area (square km)	1.000	(1.000–1.000)	0.448	1.000	(1.000–1.000)	0.001	1.000	(1.000–1.000)	0.002
Number of VNAs per 100,000 population	0.991	(0.978–0.999)	0.113	0.997	(0.991–1.000)	0.114	1.000	(0.993–1.005)	0.962
Number of clinics per 100,000 population	1.004	(1.001–1.007)	0.012	1.003	(1.001–1.006)	0.008	1.000	(0.994–1.005)	0.962
B. Organizational factor									
For-profit organization (ref = non-profit)	1.132	(0.689–1.914)	0.634	0.845	(0.582–1.242)	0.384	0.777	(0.251–1.969)	0.625
Medical organization (ref = non-medical)	0.612	(0.330–1.137)	0.118	1.050	(0.691–1.598)	0.821	0.869	(0.610–1.240)	0.437
Years of operation as of FY2014	0.938	(0.761–1.157)	0.552	0.983	(0.942–1.025)	0.427	0.938	(0.866–1.009)	0.101
Provision of care management services (ref = no)	1.117	(0.814–1.533)	0.493	0.955	(0.721–1.270)	0.748	0.724	(0.461–1.179)	0.177
Number of medical institutions issuing home-visit nursing orders	0.986	(0.945–1.029)	0.005	0.992	(0.980–1.004)	0.223	0.994	(0.976–1.012)	0.534
Number of FTE nurses	1.003	(0.970–1.035)	0.110	0.952	(0.927–0.976)	0.006	0.848	(0.755–0.942)	0.004
Percentage of nurses with less than three years of experience	1.001	(0.998–1.005)	0.493	1.002	(0.997–1.006)	0.423	0.996	(0.989–1.003)	0.297
Percentage of full-time nurses	0.996	(0.990–1.002)	0.162	1.000	(0.995–1.005)	0.934	1.005	(0.998–1.012)	0.161
Number of users per FTE nurses	1.003	(0.970–1.035)	0.878	0.952	(0.927–0.976)	<0.001	0.978	(0.944–1.011)	0.204
Change in number of nurses in 2013									
Decrease (ref = no change)	0.808	(0.361–1.656)	0.613	0.806	(0.540–1.178)	0.536	1.308	(0.845–1.985)	0.132
Increase (ref = no change)	0.955	(0.676–1.342)	0.793	0.825	(0.601–1.125)	0.603	0.887	(0.545–1.404)	0.280
Percentage of rehabilitation professionals among home-visit staff	1.002	(0.997–1.006)	0.463	1.003	(0.999–1.007)	0.101	0.997	(0.988–1.005)	0.423
Provision system of services									
Emergency home-visits (ref = no)	0.801	(0.493–1.303)	0.370	1.061	(0.669–1.706)	0.804	1.869	(1.023–3.571)	0.049
Home-based palliative care (ref = no)	0.898	(0.569–1.429)	0.645	0.814	(0.526–1.274)	0.360	0.847	(0.489–1.511)	0.564
Specialized medical care 1 (ref = no)	1.213	(0.661–2.251)	0.535	1.033	(0.552–1.949)	0.920	1.053	(0.437–2.630)	0.911
Specialized medical care 2 (ref = no)	0.802	(0.457–1.417)	0.444	0.923	(0.520–1.670)	0.787	0.531	(0.236–1.234)	0.132
Enhanced service delivery (ref = no)	1.457	(0.860–2.413)	0.151	0.728	(0.522–1.010)	0.059	0.703	(0.481–1.036)	0.071
Cooperative discharge guidance with hospitals (ref = no)	0.760	(0.531–1.091)	0.134	0.824	(0.599–1.142)	0.241	0.925	(0.608–1.431)	0.720
Number of types of medical care that can be provided	0.986	(0.945–1.029)	0.521	0.940	(0.894–0.988)	0.015	0.937	(0.873–1.006)	0.071
Percentage of users under the age of 40 years	1.019	(1.005–1.034)	0.007	1.011	(0.995–1.026)	0.174	0.968	(0.927–1.004)	0.110
Percentage of users with care need level of four or higher	1.004	(0.998–1.011)	0.202	1.003	(0.996–1.011)	0.363	0.995	(0.984–1.006)	0.397

AOR: adjusted odds ratios 95%. CI: 95% confidence interval. *p*: *p*-value.

## Data Availability

Not applicable.

## Appendix A

**Table A1:** A bivariate analysis showing the factors related to the closure of home-visit nursing agencies by years of operation.

Independent Variables	<3 Years of Operation			3–14 Years of Operation			≥15 Years of Operation		
	Not Closed	Closed	*p*	Not Closed	Closed	*p*	Not Closed	Closed	*p*
	n (%)	n (%)		n (%)	n (%)		n (%)	n (%)	
A. Regional factors									
Percentage of older adults 65 years old or over ^†^	25.7 (22.9–30.3)	25.0 (22.3–29.3)	0.007 ^b^	26.6 (23.4–30.6)	26.3 (23.6–30.6)	0.714 ^b^	27.5 (24.3–32.2)	27.1(24.4–32.3)	0.788 ^b^
Inhabitable area (square km) ^†^	2827.7 (1097.3–7898.3)	3269.3 (1083.7–8172.1)	0.330 ^b^	2488.1 (883.6–7875.3)	3669.3 (983.5–8792.5)	0.034 ^b^	1507.4 (647.1–5694.3)	1792.9 (614.4–7975.2)	0.377 ^b^
Number of VNAs per 100,000 population ^†^	7.0 (5.3–9.5)	6.5 (4.9–9.2)	0.038 ^b^	7.3 (5.3–9.4)	7.4 (5.5–10.1)	0.253 ^b^	6.8 (4.9–9.6)	7.4 (5.8–11.6)	0.004 ^b^
Number of clinics per 100,000 population ^†^	77.3 (63.5–91.5)	75.9 (63.4–92.5)	0.789 ^b^	78.6 (65.8–94.1)	81.2 (68.0–99.3)	0.012 ^b^	77.3 (64.9–91.5)	80.8 (68.0–95.6)	0.062 ^b^
B. Organizational factors									
Ownership 1									
For-profit	798 (75.7)	256 (24.3)	0.099 ^a^	1287 (87.3)	188 (12.8)	0.446 ^a^	60 (92.3)	5 (7.7)	0.774 ^a^
Non-profit	338 (79.7)	86 (20.3)		896 (88.3)	119 (11.7)		2295 (93.2)	167 (6.8)	
Ownership 2									
Medical	205 (82.7)	43 (17.3)	0.018 ^a^	464 (87.7)	65 (12.3)	0.974 ^a^	1208 (92.7)	95 (7.3)	0.318 ^a^
Non-medical	931 (75.7)	299 (24.3)		1719 (87.7)	242 (12.3)		1147 (93.7)	77 (6.2)	
Years of operation as of 2014 ^†^	1.5 (0.8–2.2)	0.9 (0.6–1.9)	<0.001 ^b^	7.9 (4.9–10.9)	6.9 (4.3–10.2)	0.002 ^b^	17.0 (15.1–19.1)	16.2 (15.2–18.1)	0.013 ^b^
Provision of care management services									
No	599 (74.5)	205 (25.5)	0.018 ^a^	739 (85.9)	121 (14.1)	0.055 ^a^	236 (90.1)	26 (9.9)	0.034 ^a^
Yes	538 (79.7)	137 (20.3)		1444 (88.6)	186 (11.4)		2119 (93.6)	146 (6.5)	
Number of medical institutions issuing home-visit nursing orders ^†^	10.0 (3.0–22.0)	4.5 (1.0–13.0)	<0.001 ^b^	22.0 (12.0–37.0)	16.0 (7.0–31.0)	<0.001 ^b^	22.0 (13.0–33.0)	16.0 (7.0–27.5)	<0.001 ^b^
Number of FTE nurses s ^†^	3.1(2.6–4.0)	3.0 (2.6–3.8)	0.003 ^b^	4.0 (3.0–5.5)	3.3 (2.8–4.9)	<0.001 ^b^	4.9 (3.6–6.8)	3.9 (3.0–5.0)	<0.001 ^b^
Percentage of nurses with less than 3 years of experience in the VNA ^†^	66.7 (75.0)	77.5 (83.4)	0.371 ^b^	33.0 (45.7)	40.0 (54.2)	0.241 ^b^	25.0 (11.1–40.0)	25.0 (0.0–40.0)	0.232 ^b^
Percentage of full-time nurses ^†^	66.7 (42.9–100.0)	66.7 (40.0–100.0)	0.802 ^b^	60.0 (40.0–80.0)	60.0 (40.0–80.0)	0.993 ^b^	66.7 (50.0–85.7)	75.0 (50.0–100.0)	0.012 ^b^
Number of users per FTE nurses ^†^	7.2 (2.6–4.0)	3.7 (0.8–9.3)	<0.001 ^b^	12.9 (9.1–18.5)	10.3 (6.1–15.8)	<0.001 ^b^	13.1 (10.0–16.8)	11.7 (7.3–15.8)	<0.001 ^b^
Change in number of nurses in fiscal 2013									
Decrease	55 (83.3)	11 (16.7)	0.037 ^a^	342 (89.3)	41 (10.7)	0.169 ^a^	441 (92.9)	34 (7.2)	0.039 ^a^
No change	707 (74.8)	238 (25.2)		1106 (86.5)	173 (13.5)		1345 (92.4)	111 (7.6)	
Increase	375 (80.1)	93 (19.9)		735 (88.8)	93 (11.2)		569 (95.5)	27 (4.5)	
Percentage of rehabilitation professionals among home-visit staff ^†^	0.0 (0.0–28.6)	0.0 (0.0–8.0)	<0.001 ^b^	4.7 (0.0–44.4)	0.0 (0.0–27.1)	<0.001 ^b^	5.0 (0.0–25.0)	0.0 (0.0–15.2)	<0.001 ^b^
Provision system of services									
Emergency home-visits									
No	249 (65.5)	131 (34.5)	<0.001 ^a^	309 (82.4)	66 (17.6)	<0.001 ^a^	234 (90.4)	25 (9.7)	0.055 ^a^
Yes	888 (80.8)	211 (19.2)		1874 (88.6)	241 (11.4)		2121 (93.5)	147 (6.5)	
Home-based palliative care									
No	338 (67.6)	162 (32.4)	<0.001 ^a^	361 (80.6)	87 (19.4)	<0.001 ^a^	259 (87.5)	37 (12.5)	<0.001 ^a^
Yes	799 (81.6)	180 (18.4)		1822 (89.2)	220 (10.8)		2096 (94.0)	135 (6.1)	
Specialized medical care 1									
No	286 (66.8)	142 (33.2)	<0.001 ^a^	237 (79.8)	60 (20.2)	<0.001 ^a^	112 (84.2)	21 (15.8)	<0.001 ^a^
Yes	851 (81.0)	200 (19.0)		1946 (88.7)	247 (11.3)		2243 (93.7)	151 (6.3)	
Specialized medical care 2									
No	292 (66.1)	150 (33.9)	<0.001 ^a^	257 (79.8)	65 (20.2)	<0.001 ^a^	110 (82.7)	23 (17.3)	<0.001 ^a^
Yes	845 (81.5)	192 (18.5)		1926 (88.8)	242 (11.2)		2245 (93.8)	149 (6.2)	
Enhanced service delivery system									
No	999 (76.1)	313 (23.9)	0.061 ^a^	1275 (85.2)	222 (14.8)	<0.001 ^a^	457 (88.7)	58 (11.3)	<0.001 ^a^
Yes	138 (82.6)	29 (17.4)		908 (91.4)	85 (8.6)		1898 (94.3)	114 (5.7)	
Cooperative discharge guidance with hospitals									
No	411 (70.0)	176 (30.0)	<0.001 ^a^	507 (82.3)	109 (17.7)	<0.001 ^a^	404 (89.0)	50 (11.0)	<0.001 ^a^
Yes	726 (81.4)	166 (18.6)		1676 (89.4)	198 (10.6)		1951 (94.1)	122 (5.9)	
Number of types of medical care that can be provided ^†^	11 (6–13)	9 (1–13)	<0.001 ^b^	12 (10–13)	11 (8–13)	<0.001 ^a^	12 (11–13)	11 (9–13)	<0.001 ^b^
Percentage of users under the age of 40 ^†^	0.0 (0.0–2.8)	0.0 (0.0–4.2)	0.977 ^b^	1.2 (0.0–4.6)	0.0 (0.0–4.5)	0.052 ^b^	2.0 (0.0–5.0)	0.0 (0.0–3.4)	<0.001 ^b^
Percentage of users with care need level of 4 or higher ^†^	31.8 (20.0–44.4)	30.8 (18.2–50.0)	0.712 ^b^	33.3 (25.0–43.6)	34.3 (24.0–48.6)	0.596 ^b^	36.1 (27.1–46.5)	34.1 (25.4–46.2)	0.235 ^b^

†: Median (25–75 percentile). *p*: *p*-value. VNAs: home-visit nursing agencies. FTE nurses: full-time equivalent nurses. ^a^: Chi-square test. ^b^: Wilcoxon rank sum test.
